# Factors That Negatively Affect the Prognosis of Pediatric Community-Acquired Pneumonia in District Hospital in Tanzania

**DOI:** 10.3390/ijms18030623

**Published:** 2017-03-13

**Authors:** Serena Caggiano, Nicola Ullmann, Elisa De Vitis, Marzia Trivelli, Chiara Mariani, Maria Podagrosi, Fabiana Ursitti, Chiara Bertolaso, Carolina Putotto, Marta Unolt, Andrea Pietravalle, Paola Pansa, Kajoro Mphayokulela, Maria Incoronata Lemmo, Michael Mkwambe, Joseph Kazaura, Marzia Duse, Francesco Nieddu, Chiara Azzari, Renato Cutrera

**Affiliations:** 1 Respiratory Unit, Bambino Gesù Children’s Hospital, 00165 Rome, Italy; nicola.ullmann@opbg.net (N.U.); renato.cutrera@opbg.net (R.C.); 2 Department of Pediatrics, Sapienza University of Rome, Umberto I Hospital, 00161 Rome, Italy; marzia.trivelli@gmail.com (M.T.); chiaramariani23@yahoo.it (C.M.); maripod@hotmail.com (M.P.); fabianaursitti@gmail.com (F.U.); chiara.bertolaso@hotmail.it (C.B.); caro_85@hotmail.it (C.P.); marta_unolt@hotmail.it (M.U.); apietravalle@gmail.com (A.P.); pansa.paola@gmail.com (P.P.); marzia.duse@gmail.com (M.D.); 3 Department of Clinical Immunology, University of Florence and Children’s University Hospital A. Meyer, 50139 Florence, Italy; ely87_dv@hotmail.it (E.D.V.); francesco.nieddu@meyer.it (F.N.); chiara.azzari@unifi.it (C.A.); 4 Pediatric Unit, St. Gaspar Hospital, Box 12, Itigi, Tanzania; akajoroa@yahoo.com (K.M.); waursulitigi@yahoo.it (M.I.L.); mikaelimkwambe@rocketmail.com (M.M.); sereka@hotmail.it (J.K.)

**Keywords:** community-acquired pneumonia, developing countries, prevention, vaccines, molecular diagnostic, antibiotic therapy

## Abstract

Community-acquired pneumonia (CAP) is still the most important cause of death in countries with scarce resources. All children (33 months ± 35 DS) discharged from the Pediatric Unit of Itigi Hospital, Tanzania, with a diagnosis of CAP from August 2014 to April 2015 were enrolled. Clinical data were gathered. Dried blood spot (DBS) samples for quantitative real-time polymerase chain reaction (PCR) for bacterial detection were collected in all 100 children included. Twenty-four percent of patients were identified with severe CAP and 11% died. Surprisingly, 54% of patients were admitted with a wrong diagnosis, which increased complications, the need for antibiotics and chest X-rays, and the length of hospitalization. Comorbidity, found in 32% of children, significantly increased severity, complications, deaths, need for chest X-rays, and oxygen therapy. Malnourished children (29%) required more antibiotics. Microbiologically, *Streptococcus pneumonia* (*S. p.*), *Haemophilus influenza type b* (*Hib*) and *Staphylococcus aureus* (*S. a.*) were the bacteria more frequently isolated. Seventy-five percent of patients had mono-infection. Etiology was not correlated with severity, complications, deaths, oxygen demand, or duration of hospitalization. Our study highlights that difficult diagnoses and comorbidities negatively affect clinical evolution. *S. p.* and *Hib* still play a large role; thus, implementation of current vaccine strategies is needed. DBS is a simple and efficient diagnostic method for bacterial identification in countries with scarce resources.

## 1. Introduction

Pneumonia is a major cause of morbidity and mortality in children and adults in low and middle-income countries [1]. Pneumonia kills an estimated 1 million children under the age of 5 every year and accounts for 15% of deaths in preschool children, [2] with around 90% occurring in the developing world [3,4]. In low-income countries, access to medical care is often difficult and the availability of anti-microbial therapy, of routine vaccination, and of supportive treatment such as nutrition and oxygen supplementation is still scarce [5]. Of the 6.3 million children who died in their first 5 years of life in 2013 worldwide, 52% died of infectious disease, and pneumonia was responsible for at least 15% of total deaths [6]. The main risk factors for community-acquired pneumonia (CAP) include age (<1 year), malnutrition, lack of breast feeding, prematurity, immunosuppression, HIV infection, overcrowding, passive tobacco exposure, exposure to indoor air pollution, and the winter season [7,8]. Co-existing illnesses, such as malaria and diarrhea, are also important contributing factors to the increased CAP burden of disease in underdeveloped countries [9]. Because of the limited access to health care in terms of microbiological and radiological investigation, clinical symptoms are often essential to make diagnoses of pneumonia and to guide empiric treatment in countries with limited resources. For instance, tachypnea alone is considered an indication to start treatment in a child with a cough in an ambulatory setting; however, when signs of respiratory distress are present, hospitalization is suggested [10]. WHO recommends a diagnostic algorithm that takes into account clinical signs such as age-specific respiratory rates, lower chest indrawing, and several key danger signs such as cyanosis and the inability to feed [11]. An accurate etiologic diagnosis is often complicated and differentiating between bacterial and nonbacterial pneumonia is clinically difficult. Current evidence suggests severe pneumonia results from infection with multiple pathogens such as bacterial-viral, dual viral, or mycobacterial bacterial infections [1]. It has been reported that up to a third of children with pneumonia may have viral-bacterial co-infections [12]. *Streptococcus pneumoniae* (*S. p.*) and *Haemophilus influenza type b* (*Hib*) are the two principal causes of bacterial infection. These pathogens can be prevented by immunization or treated with low-cost antibiotics, but it has been estimated that only 30% of children with bacterial pneumonia receive the correct treatment. *S. p.* causes up to 18% of severe cases and 33% of deaths [13], followed by *Hib* (4% of severe episodes and 16% of deaths), and, finally, by influenza virus (7% of severe episodes and 11% of deaths). Other pathogens frequently identified are *Staphylococcus aureus* (*S. a*.), *Klebsiella pneumoniae* (*K. p*.), and *Mycobacterium tuberculosis* (*M. t*.) in endemic areas. According to diagnostic tools, it is known that chest X-rays are often unable to differentiate bacterial from viral infections because of similar radiographical findings [14], and a clinical presentation has low specificity and sensitivity. With these assumptions, microbiological confirmation seems to be essential for accurate diagnosis [5] and useful for selecting the most appropriate treatment. Among microbiological techniques, on one side, the high prevalence of nasopharyngeal colonization by potentially pathogenic bacteria limits the use of respiratory samples; on the other side, blood culture has the disadvantage of offering results after 24–48 h, and it is often impaired by pre-treatment with antibiotics. Other molecular techniques such as blood PCR (polymerase chain reaction) assays have the benefit of reducing these short comings and of identifying pneumonia etiology quickly. Dried blood spot (DBS) sampling is a simple method that does not need very large volumes of any given sample, which is essential for any pediatric test and screening of large populations, especially in countries where healthcare facilities are not readily accessible [15]. DBS has been described as easily transportable and able to identify and serotype *S. p.* at room temperature [16].

The WHO estimates that 1.5 million children continue to die every year from vaccine-preventable diseases, accounting for 17% of all under-5 deaths [17]. Particularly, *S. p.* and *Hib* are considered to be the leading cause of child pneumonia deaths worldwide and a universal use of conjugated *Hib* and pneumococcal vaccines should prevent approximately 1 million child deaths per year [18,19,20]. Delayed introduction of conjugated vaccines in developing countries is mostly attributed to high cost and lack of political will, resulting in widening vaccine schedule gaps between developed and under developed countries. While some preventable risk factors for severe lung infectious disease in developing countries have been hypothesized, the literature is still scarce on the characterization of clinical presentation, prognosis, and the microbiological definition of pediatric pneumonia in low-income countries [21]. The main purpose of our prospective study was to analyze a large population of children hospitalized with CAP in a country with limited resources and identify the principle factors that negatively affect clinical evolution. Moreover, we planned to explore infection etiology by the use of reproducible DBS samples from pediatric patients and correlate results with clinical characteristics.

## 2. Results

### 2.1. Patients

Through our prospective study, we consecutively enrolled 100 children (mean age 33 months ± 35 DS) dismissed from Itigi hospital in Tanzania with a diagnosis of pneumonia. Forty-seven percent were boys, and 36% were infants (<12 months). Twenty-nine percent were classified as malnourished, identifying malnutrition in a weight for age *Z*-score ≤ 2. The average length of hospitalization was 10 ± 6 days. Twenty-four percent of patients were identified with severe pneumonia, and 11% of all children died. Demographics and clinical characteristics of the study population are clearly described and summarized in Table 1.

### 2.2. Comorbidities

Co-morbidities were present in 32% of patients with malaria as the most common condition, followed by typhoid fever and anemia (Table 1). Co-morbidities significantly increased the severity of pneumonia, including the need for oxygen and the number of deaths, and co-morbidities were significantly associated with more complications and the need for chest radiographs (*p* < 0.05). Comorbidities did not increase the length of hospitalization. Furthermore, we observed more antibiotics administered in patients without comorbidities (*p* < 0.05).

### 2.3. Initial Diagnosis Different from Pneumonia

Surprisingly, in more than half of the hospitalized patients, pneumonia was not originally recognized at the first evaluation in the emergency department when admission was decided. The incorrect diagnosis at admission significantly increased the frequency of complications, the need for ≥2 antibiotics, and radiological investigations (*p* < 0.05). Patients with an initial wrong diagnosis did not experience more oxygen demand or a higher number of deaths. As expected though, initial misdiagnosis increased the length of hospitalization by two days.

### 2.4. Malnutrition

Children with malnutrition required more antibiotic treatment (*p* < 0.05), but they did not show significant differences from other patients in terms of severity, deaths, comorbidities, or the need for medical intervention and length of stay.

### 2.5. Wheezing

Wheezing was diagnosed in 27% of children. These patients had increased severity of pneumonia (*p* < 0.01) and incremented a need for supplemental oxygen therapy (*p* < 0.05).

### 2.6. Bacterial Infections

Table 2 describes the main microbiological findings. In 52 (62%) patients, *S. p.* was isolated; *Hi* was detected in 23 patients (27%), mainly *Hib*, as was *S. a.* in 17 of our cases (20%). Five children had *Pseudomonas aeruginosa* (*P. a*.) (6%), and in 3 patients (3%), *Salmonella typhi* (*S. t.)* was detected. Seventy-five percent of patients had mono-infection, and the remaining 25% showed bacterial co-infection, by far most frequently between *S. p.* and *Hi* (57%), followed by *S. p.* and *S. a.* (14%), *Hi* and *S. a.* (14%), *Hi* + *S. t.* (9.5%), and *S. p.* + *P. a.* (5%). Patients with pneumococcical etiology showed apparently milder pneumonia (*p* < 0.05), and pneumococcal bacterium was not isolated more frequently in patients with comorbidities. Neither *S. p.* nor *Hi*, nor even *S. a.*, in mono-infection was most frequently isolated in malnourished or younger children. Moreover, none of these bacteria was associated with more severe pneumonia, the occurrence of complications, or death. Furthermore, subjects with mono-infection did not have more oxygen demand, nor did they require more chest-X rays or longer hospital admission. Interestingly, children with *Hi* infection presented less wheezing (*p* < 0.05). Finally, *S. p.* and *Hi* in coinfection were not associated with more disease severity, number of complications, days of hospitalization, or a need for chest X-rays and oxygen therapy, but this coinfection was find more frequently in children with comorbidities (*p* < 0.01).

### 2.7. Need for Oxygen

As expected, the need for oxygen was significantly associated with a clinical diagnosis of severe pneumonia and increased in patients with negative prognosis (*p* < 0.05).

### 2.8. Age

Interestingly, it was observed that a wrong diagnosis was more common in children >36 months (*p* < 0.05). Furthermore, we observed that 73% of children who died were malnourished, and those who were not underweight were younger (≤36 months old).

### 2.9. Length of Hospitalization

The average length of hospitalization was 10 ± 6 days. In patients with severe pneumonia who showed a significant increase in deaths (*p* < 0.05), we observed a shorter length of stay. They died within the first 8 days of hospitalization.

## 3. Discussion

Pneumonia is the single biggest killer of children worldwide. We described clinical and microbiological findings in children that were dismissed with a diagnosis of pneumonia during the 9-month study period (which included rainy and dry seasons with no difference among them) in the Pediatric Unit of a Tanzanian hospital. To our knowledge, few previous studies have focused only on death and hypoxia as main outcomes, while we tried to identify the principle factors that negatively affect the clinical evolution of children hospitalized with CAP in a country with limited resources. Moreover, we planned to explore infection etiology by the use of reproducible DBS samples from pediatric patients and correlate results with clinical characteristics. We looked for those factors correlating with severe pneumonia, deaths, complications, a need for oxygen and more use of antibiotics, as well as a need for chest X-rays and a certain length of stay, among other epidemiologic factors such as age, gender, weight for age, clinical symptoms, comorbidity, and microbiological outcomes. Our data showed that co-morbidity, a need for supplemental oxygen, and clinical diagnosis of severe pneumonia were correlated to increased mortality among patients hospitalized with CAP. Interestingly, patients with severe pneumonia showed a significantly shorter length of stay because death occurred within the first eight days. This data could be explained by the absence of a pediatric intensive care unit with impossibility for patients to receive intensive treatment when needed. No differences among genders and age were observed in terms of death and severity of pneumonia, but misdiagnosis was more frequent in children ≥3 years old with the direct consequence of longer hospitalization. From our population, 73% patients that died were malnourished, and the rest of the deaths occurred in young children (≤36 months old), possibly indicating that any of the factors facilitated a negative exitus. Unexpectedly, more than half of the patients had been hospitalized with a suspected diagnosis other than CAP, and the wrong diagnosis at admission increased both radiological examinations and antibiotic therapy. In rural Africa, at the moment, most hospital visits and admissions are attended only by nurses or paramedics, and diagnoses or management decisions can only be guided by clinical signs and symptoms [22]. As is known, the specificity of a clinical presentation of CAP is poor, and the diagnosis can be often difficult or uncertain, particularly with limited diagnostic resources. It is known that overlap between different diseases such as pneumonia and malaria, the most frequent comorbidity in our population, is possible. Infection such as malaria may predispose one to acquire bacterial infection but also may clinically mimic severe pneumonia. However, malaria, as a comorbidity, did not determine the worst clinical trend of CAP. A complete training of all professional figures in charge of medical care and a deep comprehension of the interaction of different diseases are essential to improving correct diagnosis and treatment [23]. Gastroenteritis and dehydration are reported as common comorbidities that may predispose one to pneumonia [24]. In our population though, we described many other different comorbidities such as typhoid fever, measles, or clinical conditions determined by local herbs intoxication. The presence of these diseases is to be related to poor adherence to international vaccination programs and to the popularity of dangerous local traditions. We reported an increase in antibiotics administration to patients with isolated pneumonia without comorbidities, which can be explained by a quicker diagnostic classification and the immediate prescription of a broad-spectrum antibiotic. In resource-limited settings, chest X-rays are rarely used to guide clinical management of severe pneumonia [21]. However, in the case of a wrong initial assessment or clinical conditions complicated by the presence of comorbidities, a chest radiograph confirmed its utility in a differential diagnosis in difficult settings. Hypoxemia was shown to be a predictor of poor outcome, as reported from other studies [22]. As expected, low blood oxygen saturation was shown to be significantly linked to severe pneumonia and death [25,26]; moreover, the need for oxygen was increased in the presence of comorbidity and wheezing. The latter was diagnosed in a quarter of patients, especially in children with severe pneumonia, but it did not appear to be related to any specific microorganism. We speculate that the viral nature of some of these infections might have triggered wheezing symptoms. It is known that impaired children’s growth and underlying malnutrition is a major risk factor for pneumonia [27], and this is supported by our result of increased and multiple antibiotic therapy used for children with a <−2 *Z*-score. However, in our population, malnutrition, described in around 25% of patients, did not show any significant correlation with other outcomes. Our data confirmed that *S. p.*, *Hib*, and *S. a.* are the main bacteria causing pneumonia. A recent study from Gambia involving children younger than 5 years with severe pneumonia demonstrated *S. p.* to be present in 91% of lung aspirates, followed by *Hi* at 23%, and *S. a.* in 6% [27]. Besides poor prevention, which can explain the bacterial etiology for all children in limited resources countries, Gram-negative bacteria have been shown to be more common in children with malnutrition [28]. In fact, *Hi* was the most common non-pneumococcal pathogen isolate in our study. In the literature, the role of non-pneumococcal pathogens is described as an independent predictor of mortality [29]. Our study described that non-pneumococcal infections were significantly correlated with severe pneumonia possibly because those patients had also compromised a general condition, were undernourished, and had more comorbidities. The same significant increase in comorbidities were observed in cases of coinfection between *Hi* and *S. p*., probably because it is more susceptible to these bacteria. Finally, we could not identify any specific bacteria as a negative predictive factor of death. As confirmed by our study, dried blood spot (DBS) sampling is a useful and easy method for preserving and transporting blood samples. This is particularly important in developing countries where health services are not easily accessible and microbiology laboratories are rare with limited technical expertise. DBS collection can overcome the obstacles associated with the collection and storage of biological samples. DBS may be suitable for the detection of infectious pathogens by real-time PCR (RT-PCR) [15]. RT-PCR, as is known, is a sensitive and specific method, which allows for the detection of a great number of pathogens. Therefore, DBS sampling can be used for pathogen detection and allow for diagnosis. The results of our study of a large population of African children contribute towards a better understanding of the main etiological causes of pneumonia in low-income countries. Our paper together with the final results of a few ongoing studies will provide more updated and comprehensive data regarding pneumonia in children living in low- and middle-income countries [30,31]. Our data highlight poor adherence to *Hib* and pneumococcal conjugate vaccines. Currently used conjugate vaccines worldwide include the 13-valent conjugate vaccines (pneumococcal conjugate vaccine 13 (PCV13)) that have shown a substantial positive impact on the burden of pneumococcal disease. The positive effects are expected to be even more dramatic among young children in low- and middle-income countries [32]. A meta-analysis of serotypes causing CAP worldwide estimated that 49% to 88% of pneumococcal deaths in Africa and Asia are caused by serotypes covered in in PCV10 and PCV13 [33]. Since 2006, the WHO has recommended that PCV and *Hib* conjugate vaccines be included in all routine immunization programs. However, the uptake of PCV in low- and middle-income countries has been limited, in large part because of a vaccine’s elevated cost. The Global Alliance for Vaccines and Immunization (GAVI) has worked hard to accelerate the diffusion of PCV10 and PCV13 vaccines in developing countries, ensuring more affordable prices. Currently, more than 190 countries have introduced a *Hib*-containing vaccine into their national immunization program [34]. In Tanzania, the Expanded Programme on Immunization in order to reduce morbidity and mortality caused by preventable infections in children under 5 years of life started in 1975. However, in the Itigi district, where our study took place, the vaccinations for *Hib* and pneumococcal conjugate vaccine 13-valent were introduced from 2012, administering vaccines among small villages in order to make vaccinations available to all. Our results, though, still confirm a high prevalence of *S. p.* and *Hi* infections in children admitted for pneumonia; therefore, our study stresses the need for a global campaign to significantly improve the adherence to the local immunization programme.

We recognize certain limits to our research study: the laboratory test could not detect viral and fungal infections; however, considering the low prevalence of microbiological negative results, we can assume that results would not be significantly different. Moreover, we could not include HIV detection because of local healthcare organization and resources.

## 4. Materials and Methods

### 4.1. Patients

In this prospective study, we consecutively enrolled all children discharged with a diagnosis of community-acquired pneumonia from Pediatric Unit of St. Gaspar Hospital in Itigi, a rural village in Tanzania, from August 2014 to April 2015. Pneumonia was defined using WHO guidelines [11]. We classified pneumonia as severe or non-severe on the basis of clinical features, being the management based on the classification. Clinical data until discharge were gathered and analyzed.

### 4.2. Definitions

According to WHO classification [11], we defined pneumonia as characterized by the following:

- fast breathing—≥50 breaths/min in a child aged 2–11 months and ≥40 breaths/min in a child aged 1–5 years;
- lower chest indrawing;
- chest auscultation suggestive of crackles, pleural rub, bronchial breath sounds, and/or abnormal vocal resonance.

Severe pneumonia defined as difficulty in breathing or cough associated with at least one of the following:

- oxygen saturation <90% registered with pulse oximetry or central cyanosis;
- severe respiratory distress;
- signs of general impairment such as convulsions, lethargy, unconsciousness, or inability to feed;
- one or more signs of pneumonia.

### 4.3. Treatment

Initial treatment for pneumonia was represented by oral amoxicillin, 40 mg/kg per dose twice a day for 5 days.

Patients with severe pneumonia were administrated intravenous ampicillin (or benzylpenicillin) and gentamicin. Ampicillin 50 mg/kg or benzylpenicillin 50,000 U/kg IM or IV every 6 h for at least 5 days. Gentamicin 7.5 mg/kg IM or IV once a day for at least 5 days. Ceftriaxone (80 mg/kg IM or IV once daily) was used in cases of failure of first line treatment.

When staphylococcal pneumonia was suspected: cloxacillin (50 mg/kg IM or IV every 6 h) or another antistaphylococcal antibiotic, such as oxacillin, flucloxacillin, or dicloxacillin and gentamicin (7.5 mg/kg IM or IV once a day) were prescribed.

Oxygen was given for levels of oxygen saturation <90% and fluids per os or intravenously were prescribed to guarantee appropriate hydration.

### 4.4. Investigations

All patients underwent a triage assessment with clinical general evaluation in the Emergency Unit and received an initial suspected diagnosis before admission was decided. In the pediatric ward, they received a new clinical evaluation. Oxygen saturation was measured with pulse oximetry in all children and blood samples taken. Blood cultures were reserved only on cerebrospinal fluid in suspected meningitis. When clinically indicated, a chest X-ray was performed.

Weight for-age *Z*-score was calculated using the 2006 WHO Child Growth Standards. Malnutrition was defined when these parameter was below −2 *Z*-score.

Drops of blood of each patient were collected on filter paper.

We used 4 punches of each sample and the DNA extraction was processed using a MagCore^®^ Automated Nucleid Acid Extractor and following MagCore^®^ Nucleid Acid Tissue Extraction Kit Protocol (Company Diatech Lab Line, Ancona, Italy). The eluate volume was 60 uL. Real-time PCR on DBS made for detecting at the beginning *S. p.*, *Hi*, *Neisseria meningitidis*, *Pneumocistis jirovecii*, *S. a.*, *S. p.* and other bacterial microorganism present in the sample, like *P. a.*, *Klebsiella pneumoniae*, and *S. t*. When we had a positive sample for *S. p.* or *Hi* detection, we proceeded with serotyping. Diagnosis of laboratory-confirmed bacteremic pneumonia was performed in the presence of positive RT-PCR for target gene, like lytA for *S. p.* or ctrA for *Neisseria meningitides*. The cycle threshold (*C*_t_) value is the PCR cycle number (of 45) at which the measured fluorescent signal exceeds as calculated background threshold, identifying the amplification of target sequence. The sample was assumed negative if there was no increase in fluorescent signal before the 40th cycle. The serotyping of *S. p.* by RT-PCR was performed using 36 different primers/probes, which were devised in a different region of the CpsA gene. We included PCV-13 serotypes and non-vaccine serotypes, according to Hsu et al [35].

### 4.5. Ethics Statement

The study, identification code SG012014, was approved on 30 July 2014 by the St. Gaspar Medical Centre Research Ethics Committee, Singida, Manyoni district, Tanzania. Informed consent was obtained for eligible children from parents or guardians.

### 4.6. Statistical Analysis

Data are expressed as a mean standard deviation. Analysis was performed using Epi Info version 3.01 (CDC, Atlanta, GA, USA). Categorical data were compared using χ^2^ test or Fisher’s test, respectively, if all expected values were <5 or ≥5. A *p*-value < 0.05 was considered significant. The Student’s *t*-test was used to compare mean values of normally distributed quantitative variables.

## 5. Conclusions

Our study confirms that pneumonia, often with the presence of comorbidities, continues to be one of the leading killers of young children in poor developing countries. Malnutrition and young age are still dangerous factors that predispose children to death. Considering the high prevalence of wrong diagnosis at admission, a complete training programme of all health workers and a standardization of treatments to guarantee the best diagnostic and therapeutic approaches is needed. Direct positive consequences include a significant reduction of disease complications and a reduction in the use of scarce healthcare resources. Moreover, we reported that the majority of severe pneumonia and deaths are still caused by *S. p.* and *Hi*. despite the actual vaccination program. More effort is therefore needed to prevent infections, to improve adherence to available vaccines, and to implement pathogen-specific vaccines into the national immunization programs. In this setting, improving the etiological surveillance of pneumonia with simple techniques such as real-time PCR from blood spots may have critical implications for prevention and treatment policies. Finally, our data still sadly confirms the discrepancies between health assistance among developed and developing countries.

## Figures and Tables

**Table 1:** Demographics and clinical characteristics of the study population.

Background Characteristics (*n* = 100 Patients)	(%)
Median age in months	33 ± 35
0–6 months	21
7–11 months	15
12–48 months	43
49–144 months	21
Boys	47
Underweight (weigh for age *Z*-score < −2)	29
**Clinical Characteristics**	
Fever	96
Cough	97
Tachypnea	35
Chest indrawing	27
Crackles	99
Decreased breath sounds	49
Hypoxia (SpO_2_ < 90%)	19
Wheezing	27
Pleural effusion or empyema	9
Severe pneumonia	24
Death	11
**Hospitalization**	
Admitting diagnosis different from pneumonia	53
Length of stay (day)	10 ± 6
Oxygen therapy	19
Chest X-ray	25
≥2 antibiotics	88
**Comorbidities (*n* = 32 Patients)**	32
Malaria	28
Typhoid fever	16
Anemia	13
Meningitis	6
Herbal poisoning	6
Osteomyelitis	6
Measles	6
Nephrotic syndrome	6
Rheumatic heart disease	6
Tuberculosis	6
Urinary tract infection	6
Down syndrome	6
Laryngospasm	6
Bowel obstruction	6
Head injury	6

**Table 2:** Description of microbiological findings.

Microbiology Findings Real-Time PCR on Dried Blood Spot Sample (*n* = 100 Patients)	(%)
Negative	16
Monoinfection	63
Coinfection	21
*Streptococcus pneumoniae*	52
*Haemophilus influenzae*	23
*H. influenzae type b*	17
*Staphylococcus aureus*	17
*Pseudomonas aeruginosa*	5
*Salmonella typhi*	3
**Coinfection**	
*Streptococcus pneumoniae* + *Haemophilus influenzae*	12
*Streptococcus pneumoniae* + *Staphylococcus aureus*	3
*Haemophilus influenzae* + *Staphylococcus aureus*	3
*Haemophilus influenzae* + *Salmonella typhi*	2
*Streptococcus pneumoniae* + *Pseudomonas aeruginosa*	1

## Abbreviations

CAP	Community-acquired pneumonia
DBS	Dried blood spot
PCR	Polymerase chain reaction
RT-PCR	Real-time polymerase chain reaction
HIV	Human immunodeficiency virus
PCV13	Pneumococcal conjugate vaccine 13
*S. p.*	*Streptococcus pneumoniae*
*Hi*	*Haemphilus influenza*
*Hib*	*Haemophilus influenza type b*
*S. a.*	*Staphilococcus aureus*
*P. a.*	*Psedomonas aeruginosa*
*S. t.*	*Salmonella typhi*
